# How does the sexual, physical and mental health of young adults not in education, employment or training (NEET) compare to workers and students?

**DOI:** 10.1186/s12889-021-10229-6

**Published:** 2021-02-26

**Authors:** Clare Tanton, Lorraine McDonagh, Melissa Cabecinha, Soazig Clifton, Rebecca Geary, Greta Rait, John Saunders, Jackie Cassell, Chris Bonell, Kirstin R. Mitchell, Catherine H. Mercer

**Affiliations:** 1 Faculty of Public Health & Policy, London School of Hygiene & Tropical Medicine, 15-17 Tavistock Place, London, WC1H 9SH UK; 2 Research Department of Primary Care and Population Health, University College London, London, UK; 3 Institute for Global Health, University College London, London, UK; 4 National Institute for Health Research Health Protection Research Unit (NIHR HPRU) in Blood Borne and Sexually Transmitted Infections at University College London in partnership with Public Health England (PHE), in collaboration with London School of Hygiene & Tropical Medicine, London, UK; 5 Department of Primary Care and Public Health Medicine, Brighton and Sussex Medical School, University of Brighton, Brighton, UK; 6 MRC/CSO Social & Public Health Sciences Unit, University of Glasgow, Glasgow, UK

**Keywords:** Not in education, employment or training (NEET), Sexual health, Cross-sectional survey, Clustering

## Abstract

**Background:**

Syndemic theory highlights the potential for health problems to interact synergistically, compounding impact. Young adults not in education, employment or training (NEET) are more likely to experience disadvantage and poorer general health outcomes. However, there is little research on their sexual health, or the extent to which this clusters with mental and physical health outcomes.

**Methods:**

Analysis of data from 16 to 24 year olds (1729 men, 2140 women) interviewed 2010–12 for Britain’s third National Survey of Sexual Attitudes and Lifestyles. Natsal-3 is a national probability sample survey using computer-assisted personal interviewing with computer-assisted self-interviewing. Participants were classified as workers, students or NEET. We used multivariable logistic regression to examine associations between being NEET (relative to worker or student) and risk behaviours and outcomes in physical, sexual and mental health domains. We then examined how risk behaviours and poor health outcomes cluster within and across domains.

**Results:**

15% men and 20% women were NEET; 36% men and 32% women were workers; and 49% men and 48% women were students. Young people who were NEET were more likely to report smoking and drug use (men) than other young people. There were few differences in sexual health, although NEETs were more likely to report condomless sex, and NEET women, unplanned pregnancy (past year). Risk behaviours clustered more within and across domains for NEET men. Among NEET women, poor health outcomes clustered across mental, physical and sexual health domains.

**Conclusions:**

Harmful health behaviours (men) and poor health outcomes (women) clustered more in those who are NEET. This points to a possible syndemic effect of NEET status on general ill health, especially for women. Our paper is novel in highlighting that elevated risk pertains to sexual as well as mental and physical health.

**Supplementary Information:**

The online version contains supplementary material available at 10.1186/s12889-021-10229-6.

## Background

Early adulthood is a period of key transitions and a time when sexually-intimate relationships are first established. This raises a number of challenges regarding physical, psychological, and sexual health. Young adults are at higher risk of poor sexual health outcomes including sexually transmitted infections (STIs), unplanned pregnancy and sexual violence [1–4]. This life-stage is when patterns of risk behaviours begin, for example, in terms of substance use [5, 6]. It is also the time when common mental health disorders (e.g. depression) can manifest, [7] particularly among young women [8]. Death by accident is also higher, reflecting greater risk-taking at this time of life [9].

Transitions to adulthood are becoming more protracted [10]. One subgroup of young people who have been the focus of much attention are those ‘not in education, employment or training’ (NEET) [11]. In the UK in 2017, 11.1% of 16–24 year olds were in this position [12]. However, the NEET population fluctuates considerably with 35% of 19 year-olds ever having been NEET and 14% having been NEET at one point for more than a year [13]. The NEET population is heterogeneous, including young adults who are vulnerable and those who are not, with subgroups including: unemployed, unavailable, disengaged, opportunity-seekers, and voluntary [11]. However, young people in this group are not accumulating skills and social bonding through the formal channels of education, training or employment, [14] placing them at risk of future poor employment outcomes [10] and social exclusion [11]. Not being in education, employment or training is more likely to be experienced by those already disadvantaged (e.g. having a disability, having been suspended/excluded from school, substance abuse [12], low education level [15] and those with health problems/disability [11, 16]) and those from families of lower socio-economic status, lower education levels and long-term unemployed [15, 17] and this group is therefore a proxy for disadvantaged young people. Moreover, the consequences are long-term, with being NEET in young adulthood associated with future labour-market exclusion [18].

Previous research has demonstrated a higher prevalence of mental health problems in those who are NEET, [19] with depression, alcohol or substance misuse and suicidal attempts increased in economically-inactive young adults compared to student peers [20]. Adolescent depression reduces quality of life, is a risk factor for suicide, and often worsens physical and sexual health [21]. Mental health problems at this life-stage also have long-reaching impact, including substance misuse and suicide risk later in life. Moreover, many key determinants of general health and well-being, such as alcohol and drug misuse, smoking, and mental health are associated with sexual risk behaviours and poor sexual health outcomes [22, 23].

The Syndemic Model of Health suggests that rather than diseases being separate from others, and independent of social context, that there is a synergistic interaction between diseases and their social context which impacts on both individual and societal health. The theory suggests that there is the potential for a compounding of inequalities, further exacerbated by prior social disadvantage and risk of social exclusion, particularly where harmful health behaviours cluster within individuals [24].

Despite the prevalence of poor sexual health outcomes in young adults, [1–4] little is known about the sexual health needs of NEETs in particular, as disadvantaged young people, so an aim of this paper is to use the rich sexual behaviour and outcome data from Britain’s third National Survey of Sexual Attitudes and Lifestyles (Natsal-3) to examine sexual risk and outcomes, alongside data on physical and mental health outcomes to determine how risk behaviours (including substance use and sexual behaviours) and health outcomes (physical, sexual and mental) reported by young people who are NEET compare to those reported by other young people (those in employment or education). Drawing on syndemic theory, we then explore the extent to which risk behaviours and poor health outcomes cluster both within and across these health domains for NEETs vs. workers and students, to capture the extent to which these inequalities are heightened for NEETs.

## Methods

Natsal-3 was a multistage, stratified, clustered probability sample of men and women aged 16–74 in Britain. A total of 15,162 people were interviewed between September 2010 and August 2012. The response rate was 57.7%. Full details of the methodology have been published elsewhere [25, 26]. Participants were interviewed in their homes by professional interviewers via a computer-administered personal interview (CAPI) and a computer-assisted self-interview (CASI).

### Study population

The present analysis was restricted to participants aged 16–24 (1729 men and 2140 women). We used the question in Table 1 (asked in the CAPI) to assign individuals to one of three groups: in education or training (hereafter referred to as students/trainees), in employment (hereafter referred to as workers) and NEETs.

**Table 1:** Questions used to determine appropriate grouping of young people

*‘Which of these descriptions applies to what you were doing last week, that is, in the seven days ending last Sunday?’ Code all that apply*	
*1. Going to school or college full-time (including on vacation)*	
*2. In paid employment or self-employed (or temporarily away)*	
*3. On a Government scheme for employment or training*	
*4. Doing unpaid work for a business that you own, or that a relative owns*	
*5. Waiting to take up paid work already obtained*	
*6. Looking for paid work or a Government training scheme (unemployed)*	
*7. Intending to look for work but prevented by temporary sickness or injury*	
*8. Permanently unable to work because of long-term sickness or disability*	
*9. Retired from paid work*	
*10. Looking after home or family*	
*11. Doing something else (specify)*	

The definitions from the Office for National Statistics were used to categorise the groups for analysis [27]. We first defined students/trainees as participants who were enrolled either in full time education (*n* = 1718), in a government employment/training scheme (*n* = 38), or had not yet finished full-time education (*n* = 97). We then defined workers as those participating in paid employment and were *not* engaged in either full-time education or a government training scheme (*n* = 1340). The remaining young adults were defined as NEET (*n* = 738), and include those who are unemployed and those economically-inactive (e.g. those with caring responsibilities). Additional file 2 gives a breakdown of this group.

### Measures

Details of the measures used in this paper are given in Additional file 3. Most data on general health and well-being were collected in the CAPI, including on alcohol consumption and smoking, self-appraisal of general health and experience of a range of chronic conditions (including treatment for depression in the past year). Participants were also asked about their age at, and the circumstances of, first heterosexual sex, using showcards so participants responded with a letter code, to increase privacy. Questions about drug use, sexual behaviours, sexual health outcomes (including non-volitional sex, STI diagnosis and distress/worry about sex life) were asked in the CASI. Along with questions about unplanned pregnancy in the past year (to women only) using a psychometrically-validated measure [28]. Following the CASI, an additional face-to-face section asked demographics including ethnicity, household structure and social class (as measured by the National Statistics Socio-Economic Classification [29]).

### Statistical analysis

To account for the stratification, clustering, and weighting of the Natsal-3 data, [26] all analyses were completed using the complex survey functions of Stata (version 14.1). We calculated descriptive statistics to compare the demographics, general and sexual health behaviours and outcomes of students, workers and NEETs. To account for differences in the age distribution of the three populations, we present age-standardised descriptive statistics. All analyses are stratified by sex reflecting gender differences in the composition of the NEET group (Additional file 2). Multivariable logistic regression was used to calculate age-adjusted odds ratios (AORs) to investigate how the reporting of health behaviours and outcomes differed for NEETs compared to i) workers, ii) students/trainees (combined owing to few trainees (*n* = 38)) and iii) a combined group of workers and students/trainees.

We then compared the clustering of risk behaviours and poor health outcomes between the three groups. We looked separately at: risk behaviours (i.e. behaviours with the potential to harm future health) and poor health outcomes (i.e. already experienced harms) categorised into mental, physical and sexual health domains. We first examined the extent to which the different population groups reported multiple risk behaviours *within* two health domains: the mental/physical health domain and the sexual health domain. We summed the number of behaviours reported (see Table 2) out of a total of three harmful mental/physical health behaviours and a total of three harmful sexual behaviours. We then used proportional Venn diagrams to examine the extent of overlap *across* health domains as the proportion reporting harmful behaviours in both health domains. We used a similar approach to look at reporting of multiple poor health outcomes within and across three domains: physical, sexual, and mental health (Table 2).

**Table 2:** Harmful health behaviours and poor health outcomes reported in Natsal-3 identified within health domains

Harmful health behaviours	Poor health outcomes
**Domain: Mental/physical health** - Current smoker - Usually drink > 6(women)/8(men) units of alcohol on one occasion (‘binge drinking’) at least weekly - Any illicit drug use, past year	**Domain: Physical health** - Current health described as ‘fair’, ‘bad’ or ‘very bad’ - Long-standing illness/disability/infirmity - 1+ chronic condition (excluding depression) - Unhealthy BMI (< 18 | > 25)
	**Domain: Mental health** - Received treatment for depression from a health professional, past year
**Domain: Sexual health** - 2+ condomless sex partners, past year - 1+ new unprotected sex partner, past year - 5+ partners, past year	**Domain: Sexual health** - STI diagnosis, past year - Unplanned pregnancy, past year (women only) - Distressed/worried about sex life, past year - Experienced completed non-volitional sex, ever

There are many statistical tests reported in this paper and some associations may arise by chance. Associations with 95% CIs close to the null value of 1 should be interpreted with caution. Results should be interpreted holistically and conclusions should not be drawn on the basis of one test result.

## Results

### Socio-demographic characteristics

Among young adults aged 16–24, just under half of males and females (49.0 and 47.8%, respectively) were defined as students, 35.7% of males and 32.3% of females were defined as workers and 15.3% of males and 19.8% of females were categorised as NEETs (Additional file 1). Additional file 2 describes the NEET population in more detail. NEET men were mostly unemployed (70%), whereas NEET women were mostly looking after the home/family (52%). These were the only categories which showed substantial gender difference.

Men who were NEET were more likely than other men to be ethnically white, have parents who are partly skilled and live in more deprived areas. Women who were NEET were also more likely to have parents who are partly skilled or unskilled and live in more deprived areas. Both men and women who were NEET were more likely to live with a partner and/or children and were more likely to be married, in a civil partnership or cohabiting with a partner. Further comparison of the three groups can be found in Additional file 1.

### Risk behaviours and physical and mental health outcomes

Young people who were NEET were more likely to report currently smoking than workers or students (Tables 3 & 4), although drinking more than recommended did not differ between the groups. NEET men (only) were more likely than students and workers to report using drugs (AOR = 1.98).

**Table 3:** **Health behaviours and outcomes of 16–24 year old men overall and by education/employment status**

	**MEN**							
	All young men(16–24)		NEETs		Workers		Students	
*Denominators (unwt, wt)*	***1729, 1238***		***270, 189***		***605, 442***		***853, 607***	
			*Age-standardised estimates:*					
Health behaviours								
**% (95%CI) current smoker**	32.2%	[29.6–34.8%]	58.2%	[51.5–64.6%]	33.9%	[29.7–38.3%]	27.3%	[22.1–33.2%]
***AOR (95% CI), comparing:***								
*NEETs with workers (ref)*			*2.79*	*(2.02–3.85)*				
*NEETs with students (ref)*			*3.67*	*(2.54–5.28)*				
*NEETs with workers and students (combined, ref)*			*3.48*	*(2.58–4.69)*				
**Average alcohol consumption per week**								
**% (95% CI:)**								
None	24.2%	[21.9–26.6%]	24.7%	[19.4–30.7%]	17.5%	[14.1–21.6%]	24.5%	[19.8–29.9%]
Not more than recommended	70.5%	[67.9–73.0%]	67.6%	[61.2–73.4%]	76.8%	[72.5–80.7%]	70.7%	[65.1–75.7%]
**More than recommended**	5.3%	[4.2–6.6%]	7.7%	[4.9–12.0%]	5.6%	[4.0–8.0%]	4.8%	[3.1–7.3%]
***AOR (95% CI), comparing:***								
*NEETs with workers (ref)*			*1.41*	*(0.76–2.60)*				
*NEETs with students (ref)*			*1.45*	*(0.71–2.95)*				
*NEETs with workers and students (combined, ref)*			*1.53*	*(0.87–2.70)*				
**Drug use in last year**								
**% (95% CI:)**								
No	64.6%	[61.9–67.2%]	51.3%	[44.6–57.9%]	65.7%	[61.0–70.0%]	68.5%	[62.7–73.8%]
**Cannabis only**	22.6%	[20.3–25.1%]	27.7%	[21.8–34.5%]	21.1%	[17.4–25.4%]	19.8%	[15.7–24.7%]
**Cannabis and/or other drugs**	12.8%	[11.0–14.8%]	21.1%	[15.8–27.5%]	13.2%	[10.4–16.6%]	11.7%	[8.0–16.9%]
***AOR (95% CI), comparing:***								
*NEETs with workers (ref)*			*1.81*	*(1.31–2.52)*				
*NEETs with students (ref)*			*2.17*	*(1.51–3.12)*				
*NEETs with workers and students (combined, ref)*			*1.98*	*(1.46–2.67)*				
Health outcomes								
**% (95% CI) self-reported health status (fair/bad/very bad)**	8.2%	[6.9–9.7%]	18.8%	[14.0–24.7%]	7.1%	[5.1–9.9%]	4.6%	[3.1–6.8%]
***aAOR (95% CI), comparing:***								
*NEETs with workers (ref)*			*3.17*	*(1.90–5.31)*				
*NEETs with students (ref)*			*3.77*	*(2.41–5.90)*				
*NEETs with workers and students (combined, ref)*			*3.39*	*(2.24–5.13)*				
**% (95% CI) longstanding illness, disability or infirmity**	13.7%	[11.9–15.7%]	17.7%	[13.0–23.5%]	10.9%	[8.5–13.8%]	15.7%	[11.9–20.5%]
***AOR (95% CI), comparing:***								
*NEETs with workers (ref)*			*1.81*	*(1.15–2.87)*				
*NEETs with students (ref)*			*1.15*	*(0.73–1.82)*				
*NEETs with workers and students (combined, ref)*			*1.42*	*(0.96–2.12)*				
**% (95% CI) 1+ chronic health condition** ^**a**^	6.8%	[5.6–8.2%]	6.5%	[4.2–9.9%]	7.2%	[5.2–10.0%]	6.3%	[4.2–9.3%]
***AOR (95% CI), comparing:***								
*NEETs with workers (ref)*			*0.89*	*(0.49–1.62)*				
*NEETs with students (ref)*			*0.97*	*(0.54–1.74)*				
*NEETs with workers and students (combined, ref)*			*0.91*	*(0.54–1.53)*				
**Body-mass index**								
**% (95% CI)**								
Normal: 18.5–25 kg/m3	66.0%	[63.3–68.6%]	60.7%	[53.4–67.5%]	64.7%	[60.3–68.9%]	68.2%	[62.6–73.3%]
**Underweight: < 18.5 kg/m3**	6.0%	[4.8–7.6%]	8.4%	[4.9–14.2%]	1.9%	[1.0–3.7%]	8.3%	[5.5–12.4%]
**Overweight: 25–30 kg/m3**	20.5%	[18.3–22.8%]	21.1%	[15.9–27.4%]	24.9%	[21.1–29.0%]	17.6%	[13.5–22.6%]
**Obese: > 30 kg/m3**	7.5%	[6.1–9.1%]	9.8%	[6.3–15.1%]	8.5%	[6.4–11.2%]	5.9%	[3.6–9.4%]
***AOR (95% CI), comparing:***								
*NEETs with workers (ref)*			*1.24*	*(0.87–1.76)*				
*NEETs with students (ref)*			*1.32*	*(0.90–1.92)*				
*NEETs with workers and students (combined, ref)*			*1.24*	*(0.89–1.71)*				
**% (95%CI) treated for depression in the past year**	3.0%	[2.2–3.9%]	7.7%	[5.1–11.5%]	2.0%	[1.1–3.6%]	2.0%	[1.1–3.5%]
***AOR (95% CI), comparing:***								
*NEETs with workers (ref)*			*4.30*	*(1.93–9.56)*				
*NEETs with students (ref)*			*2.88*	*(1.47–5.62)*				
*NEETs with workers and students (combined, ref)*			*3.57*	*(1.98–6.46)*				

Abbreviations: unwt, unweighted; wt, weighted

% and 95% CIs are age standardised

Age-adjusted odds ratio for reporting the responses in bold font (for those variables with > 2 response options) relative to workers, relative to students and relative to both workers and students combined

^a^ Chronic health condition includes arthritis, heart attack, coronary heart disease, angina, other forms of heart disease, hypertension, stroke, diabetes, broken hip or pelvis bone or hip replacement ever, backache lasting longer than 3 mo, any other muscle or bone disease lasting longer than 3 mo, treatment for depression, treatment for cancer, and treatment for any thyroid condition in the past year

**Table 4:** Health behaviours and outcomes of 16–24 year old women overall and by education/employment status

	**WOMEN**							
	All young women(16–24)		NEETs		Workers		Students	
*Denominators (unwt, wt)*	***2140, 1207***		***468, 239***		***670, 390***		***999, 577***	
			*Age-standardised estimates:*					
Health behaviours								
**% (95%CI) current smoker**	29.6%	[27.6–31.8%]	46.3%	[41.3–51.4%]	37.1%	[33.0–41.4%]	21.3%	[17.2–26.2%]
***AOR (95% CI), comparing:***								
*NEETs with workers (ref)*			*1.47*	*(1.11–1.95)*				
*NEETs with students (ref)*			*3.76*	*(2.73–5.18)*				
*NEETs with workers and students (combined, ref)*			*2.37*	*(1.84–3.06)*				
**Average alcohol consumption per week**								
**% (95% CI:)**								
None	34.2%	[31.9–36.7%]	46.9%	[41.8–52.1%]	26.1%	[22.5–30.2%]	33.2%	[27.4–39.5%]
Not more than recommended	53.2%	[50.8–55.7%]	41.4%	[36.5–46.4%]	58.2%	[53.9–62.5%]	49.9%	[44.0–55.8%]
**More than recommended**	12.5%	[11.0–14.2%]	11.7%	[8.8–15.5%]	15.6%	[12.7–19.0%]	16.9%	[12.8–22.1%]
***AOR (95% CI), comparing:***								
*NEETs with workers (ref)*			*0.72*	*(0.48–1.09)*				
*NEETs with students (ref)*			*0.68*	*(0.40–1.14)*				
*NEETs with workers and students (combined, ref)*			*0.81*	*(0.55–1.19)*				
**Drug use in last year**								
**% (95% CI:)**								
No	80.8%	[78.7–82.7%]	79.5%	[75.1–83.3%]	79.7%	[75.9–83.0%]	75.4%	[69.3–80.7%]
**Cannabis only**	11.9%	[10.4–13.7%]	12.5%	[9.5–16.1%]	11.2%	[8.7–14.3%]	15.7%	[11.3–21.5%]
**Cannabis and/or other drugs**	7.3%	[6.1–8.6%]	8.0%	[5.6–11.5%]	9.2%	[6.9–12.1%]	8.8%	[5.9–13.0%]
***AOR (95% CI), comparing:***								
*NEETs with workers (ref)*			*1.08*	*(0.77–1.52)*				
*NEETs with students (ref)*			*0.92*	*(0.63–1.35)*				
*NEETs with workers and students (combined, ref)*			*1.14*	*(0.84–1.53)*				
Health outcomes								
**% (95% CI) self-reported health status (fair/bad/very bad)**	10.9%	[9.6–12.4%]	20.7%	[16.9–25.1%]	9.0%	[6.8–11.7%]	7.6%	[5.3–10.9%]
***AOR (95% CI), comparing:***								
*NEETs with workers (ref)*			*2.76*	*(1.87–4.07)*				
*NEETs with students (ref)*			*2.82*	*(1.88–4.23)*				
*NEETs with workers and students (combined, ref)*			*2.82*	*(2.04–3.91)*				
**% (95% CI) longstanding illness, disability or infirmity**	17.5%	[15.8–19.4%]	24.8%	[20.3–29.9%]	14.2%	[11.6–17.3%]	18.8%	[14.5–24.0%]
***AOR (95% CI), comparing:***								
*NEETs with workers (ref)*			*2.04*	*(1.44–2.89)*				
*NEETs with students (ref)*			*1.37*	*(0.96–1.95)*				
*NEETs with workers and students (combined, ref)*			*1.68*	*(1.25–2.26)*				
**% (95% CI) 1+ chronic health condition** ^**a**^	11.2%	[9.8–12.8%]	16.0%	[12.6–20.2%]	10.6%	[8.3–13.5%]	11.0%	[7.5–15.7%]
***AOR (95% CI), comparing:***								
*NEETs with workers (ref)*			*1.68*	*(1.13–2.48)*				
*NEETs with students (ref)*			*1.38*	*(0.90–2.14)*				
*NEETs with workers and students (combined, ref)*			*1.54*	*(1.11–2.15)*				
**Body-mass index**								
**% (95% CI)**								
Normal: 18.5–25 kg/m3	64.3%	[61.8–66.7%]	53.3%	[47.6–58.9%]	63.8%	[59.4–67.9%]	68.5%	[63.1–73.5%]
**Underweight: < 18.5 kg/m3**	8.8%	[7.5–10.3%]	9.0%	[6.2–13.0%]	7.1%	[4.9–10.2%]	11.6%	[8.2–16.2%]
**Overweight: 25–30 kg/m3**	17.5%	[15.8–19.5%]	23.6%	[18.9–28.9%]	18.7%	[15.6–22.3%]	13.5%	[10.5–17.2%]
**Obese: > 30 kg/m3**	9.4%	[8.1–11.0%]	14.1%	[10.9–18.2%]	10.4%	[8.1–13.3%]	6.4%	[4.1–9.8%]
***AOR (95% CI), comparing:***								
*NEETs with workers (ref)*			*1.65*	*(1.22–2.23)*				
*NEETs with students (ref)*			*1.69*	*(1.22–2.34)*				
*NEETs with workers and students (combined, ref)*			*1.72*	*(1.31–2.25)*				
**% (95%CI) treated for depression in the past year**	9.1%	[7.8–10.6%]	15.5%	[12.3–19.2%]	10.0%	[7.7–12.8%]	8.2%	[5.6–12.0%]
***AOR (95% CI), comparing:***								
*NEETs with workers (ref)*			*1.72*	*(1.14–2.58)*				
*NEETs with students (ref)*			*1.93*	*(1.22–3.05)*				
*NEETs with workers and students (combined, ref)*			*1.98*	*(1.39–2.83)*				

Abbreviations: unwt, unweighted; wt, weighted

% and 95% CIs are age standardised

Age-adjusted odds ratio for reporting the responses in bold font (for those variables with > 2 response options) relative to workers, relative to students and relative to both workers and students combined

^a^ Chronic health condition includes arthritis, heart attack, coronary heart disease, angina, other forms of heart disease, hypertension, stroke, diabetes, broken hip or pelvis bone or hip replacement ever, backache lasting longer than 3 mo, any other muscle or bone disease lasting longer than 3 mo, treatment for depression, treatment for cancer, and treatment for any thyroid condition in the past year

Young people of both sexes who were NEET reported poorer health profiles in terms of physical and mental health than students or workers (Tables 3 & 4). NEETs were more likely to report their health as ‘fair/bad/very bad’ (AOR men = 3.39, AOR women = 2.82) than students or workers, and were more likely to have a longstanding illness, disability or infirmity than workers (AOR men = 1.81, AOR women = 2.04), but there was no difference between NEETs and students despite differences in self-perceived subjective health. Women who were NEET were more likely to report chronic health condition(s) than workers. There were no differences between NEET women and students or among the three groups for men. There were few differences in BMI for men. However, women who were NEET were more likely to have a BMI either below or above the normal range (AOR = 1.72). Young people who were NEET were more likely to have received treatment for depression (past year; AOR men = 3.57, AOR women = 1.98).

### Sexual behaviour

Young people who were NEET were more likely than workers or students to report heterosexual debut before age 16 and were less likely to be sexually competent on that occasion (Tables 5 & 6). Looking at recent sexual behaviour, there was little difference in partner numbers, although NEETs were more likely than students to report sexual partner(s) over the past year. NEETs were also more likely than students to report condomless sex with 2 or more partners (past year) and were more likely than students or workers to have not used condoms for first sex with their most recent partner (AOR men = 2.19, AOR women = 1.57).

**Table 5:** Sexual behaviours, sexual health outcomes and health-seeking behaviours of 16–24 year old men overall and by education/employment status

	**MEN**							
	All young men(16–24)		NEETs			Workers	Students	
Denominators (unwt, wt)	***1729, 1238***		***270, 189***			***605, 442***	***853, 607***	
			*Age-standardised* *estimates:*					
Sexual behaviours								
**% (95%CI) First heterosexual sex before the age of 16**	31.2%	[28.8–33.8%]	45.5%	[38.5–52.6%]	38.4%	[33.9–43.1%]	19.8%	[16.0–24.2%]
***AOR (95% CI), comparing:***								
*NEETs with workers (ref)*			*1.36*	*(0.95–1.95)*				
*NEETs with students (ref)*			*3.46*	*(2.36–5.07)*				
*NEETs with workers and students (combined, ref)*			*2.16*	*(1.57–2.98)*				
**% (95%CI) Not sexually competent at first** **heterosexual sex**	43.8%	[40.7–46.9%]	58.4%	[51.4-65.2%]	44.2%	[39.5–49.0%]	38.7%	[32.2–45.6%]
***AOR (95% CI), comparing:***								
*NEETs with workers (ref)*			*1.75*	*(1.25–2.46)*				
*NEETs with students (ref)*			*2.35*	*(1.59–3.48)*				
*NEETs with workers and students (combined, ref)*			*2.01*	*(1.47–2.76)*				
**Number of partners** ^**a**^**, past year**								
**% (95% CI)**								
0	22.6%	[20.4–24.9%]	16.4%	[11.9–22.2%]	9.7%	[7.3–12.7%]	28.3%	[23.3–33.8%]
1	42.6%	[39.8–45.4%]	43.1%	[36.4–50.1%]	48.1%	[43.6–52.6%]	41.4%	[35.7–47.4%]
2	15.3%	[13.4–17.3%]	20.0%	[14.6–26.8%]	16.8%	[13.7–20.5%]	12.9%	[9.6–17.2%]
3–4	11.1%	[9.5–12.8%]	12.2%	[8.7–16.9%]	13.4%	[10.6–16.8%]	9.5%	[6.8–13.3%]
**5+**	8.5%	[7.2–10.0%]	8.3%	[5.5–12.2%]	12.0%	[9.3–15.4%]	7.9%	[5.3–11.6%]
***AOR (95% CI), comparing:***								
*NEETs with workers (ref)*			*0.62*	*(0.37–1.05)*				
*NEETs with students (ref)*			*1.16*	*(0.59–2.28)*				
*NEETs with workers and students (combined, ref)*			*0.93*	*(0.56–1.55)*				
**% (95%CI) condomless sex with 2+ partners, past year**	6.2%	[5.0–7.6%]	8.8%	[5.9–12.9%]	9.9%	[7.3–13.3%]	3.4%	[1.9–6.0%]
***AOR (95% CI), comparing:***								
*NEETs with workers (ref)*			*0.89*	*(0.51–1.55)*				
*NEETs with students (ref)*			*2.75*	*(1.34–5.63)*				
*NEETs with workers and students (combined, ref)*			*1.50*	*(0.90–2.51)*				
**% (95%CI) same-sex experience with genital contact, ever**	4.0%	[3.1-5.1%]	3.3%	[1.8–6.1%]	4.2%	[2.8–6.2%]	4.0%	[2.4–6.6%]
***AOR (95% CI), comparing:***								
*NEETs with workers (ref)*			*0.80*	*(0.37–1.74)*				
*NEETs with students (ref)*			*0.66*	*(0.29–1.50)*				
*NEETs with workers and students (combined, ref)*			*0.75*	*(0.37–1.52)*				
Characteristics of sex with most recent partner (MRP)								
**% (95%CI) condom not used on 1st occasion with** **most recent partner (vaginal/anal sex only)**	29.7%	[27.1–32.5%]	45.0%	[37.8–52.4%]	30.2%	[26.2–34.6%]	22.7%	[17.6–28.7%]
***AOR (95% CI), comparing:***								
*NEETs with workers (ref)*			*1.77*	*(1.24–2.53)*				
*NEETs with students (ref)*			*2.98*	*(1.93–4.60)*				
*NEETs with workers and students (combined, ref)*			*2.19*	*(1.56–3.08)*				
**% (95%CI) just met MRP when first had sex**	6.3%	[5.1–7.8%]	6.9%	[4.0–11.5%]	6.0%	[4.1–8.6%]	6.7%	[4.4–10.1%]
***AOR (95% CI), comparing:***								
*NEETs with workers (ref)*			*1.18*	*(0.60–2.33)*				
*NEETs with students (ref)*			*0.96*	*(0.45–2.03)*				
*NEETs with workers and students (combined, ref)*			*1.13*	*(0.60–2.11)*				
	30.6%	[27.9,33.4%]	31.7%	[25.5,38.7%]	24.4%	[20.5,28.8%]	31.7%	[26.5,37.5%]
	18.4%	[16.3,20.7%]	18.7%	[13.8,24.7%]	17.1%	[13.6,21.2%]	20.8%	[16.3,26.3%]
	11.5%	[9.7,13.5%]	8.0%	[5.1,12.3%]	11.0%	[8.3,14.4%]	13.8%	[9.9,18.9%]
	35.4%	[32.6,38.7%]	36.5%	[29.3,44.3%]	42.6%	[37.8–47.5%]	30.9%	[24.4,38.2%]
	4.0%	[2.9,5.4%]	5.2%	[2.9–9.1%]	5.0%	[3.5–7.0%]	2.7%	[0.9–8.3%]
Sexual health outcomes								
**% (95%CI) Diagnosed with STI, past year**	2.3%	[1.6-3.2%]	1.6%	[0.6 – 3.9%]	3.3%	[2.0 – 5.3%]	1.6%	[0.8 – 3.1%]
***AOR (95% CI), comparing:***								
*NEETs with workers (ref)*			*0.43*	*(0.15–1.23)*				
*NEETs with students (ref)*			*1.03*	*(0.29–3.57)*				
*NEETs with workers and students (combined, ref)*			*0.61*	*(0.21–1.73)*				
**% (95%CI) Experienced attempted non-volitional sex, ever**	4.5%	[3.4–6.0%]	3.9%	[2.0 – 7.7%]	5.5%	[3.6 – 8.2%]	3.6%	[2.3 – 5.7%]
***AOR (95% CI), comparing:***								
*NEETs with workers (ref)*			*0.74*	*(0.32–1.69)*				
*NEETs with students (ref)*			*0.93*	*(0.37–2.34)*				
*NEETs with workers and students (combined, ref)*			*0.84*	*(0.38–1.85)*				
**% (95%CI) Experienced completed non-volitional sex, ever**	1.0%	[0.6–1.8%]	0.7%	[0.1 – 3.2%]	1.6%	[0.8 – 3.3%]	0.8%	[0.3 – 2.0%]
***AOR (95% CI), comparing:***								
*NEETs with workers (ref)*			*0.45*	*(0.08–2.63)*				
*NEETs with students (ref)*			*0.78*	*(0.11–5.40)*				
*NEETs with workers and students (combined, ref)*			*0.64*	*(0.12–3.45)*				
**% (95%CI) Distressed/worried about sex life**	10.2%	[8.7–11.9%]	15.6%	[11.0, 21.5%]	13.1%	[10.4, 16.3%]	17.3%	[13.1, 22.5%]
***AOR (95% CI), comparing:***								
*NEETs with workers (ref)*			*0.97*	*(0.57–1.66)*				
*NEETs with students (ref)*			*1.12*	*(0.67–1.89)*				
*NEETs with workers and students (combined, ref)*			*1.01*	*(0.62–1.64)*				
Health seeking behaviours								
**% (95%CI) Attended sexual health clinic, past year**	16.6%	[14.5–18.9%]	20.5%	[15.0 – 27.2%]	15.7%	[12.4 – 19.6%]	16.5%	[14.4 – 18.8%]
***AOR (95% CI), comparing:***								
*NEETs with workers (ref)*			*1.41*	*(0.88–2.26)*				
*NEETs with students (ref)*			*1.33*	*(0.82–2.14)*				
*NEETs with workers and students (combined, ref)*			*1.40*	*(0.92–2.13)*				
**% (95%CI) Blood test for HIV, past year**	5.2%	[4.1–6.5%]	5.3%	[3.1 – 9.1%]	6.0%	[4.1 – 8.8%]	6.2%	[3.9 – 9.7%]
***AOR (95% CI), comparing:***								
*NEETs with workers (ref)*			*0.84*	*(0.42–1.69)*				
*NEETs with students (ref)*			*0.82*	*(0.34–1.96)*				
*NEETs with workers and students (combined, ref)*			*0.91*	*(0.47–1.77)*				
**% (95%CI) Chlamydia test, past year**	34.6%	[31.9–37.4%]	38.1%	[31.3 – 45.5%]	34.4%	[30.0 – 39.1%]	32.5%	[27.9 – 37.5%]
***AOR (95% CI), comparing:***								
*NEETs with workers (ref)*			*1.19*	*(0.82–1.72)*				
*NEETs with students (ref)*			*1.26*	*(0.85–1.86)*				
*NEETs with workers and students (combined, ref)*			*1.25*	*(0.89–1.76)*				

Abbreviations: unwt, unweighted; wt, weighted

% and 95% CIs are age standardised

Age-adjusted odds ratio for reporting the responses in bold font (for those variables with > 2 response options) relative to workers, relative to students and relative to both workers and students combined

^a^ Opposite and/or same sex partners

**Table 6:** Sexual behaviours, sexual health outcomes and health-seeking behaviours of 16–24 year old women overall and by education/employment status

	**WOMEN**							
	All young women(16–24)		NEETs		Workers		Students	
Denominators (unwt, wt)	***2140, 1207***		***468, 239***		***670, 390***		***999, 577***	
		*Age-standardised* *estimates:*						
Sexual behaviours								
**% (95%CI) First heterosexual sex before the age of 16**	29.6%	[27.4 – 31.9%]	43.4%	[38.5 – 48.4%]	33.1%	[29.4 – 37.1%]	20.5%	[16.8 – 24.8%]
***AOR (95% CI), comparing:***								
*NEETs with workers (ref)*			*1.59*	*(1.21–2.10)*				
*NEETs with students (ref)*			*3.37*	*(2.48–4.58)*				
*NEETs with workers and students (combined, ref)*			*2.36*	*(1.86–3.01)*				
**% (95%CI) Not sexually competent at firstheterosexual sex**	51.9%	[49.2 – 54.6%]	67.9%	[62.6 – 72.7%]	49.0%	[44.6 – 53.3%]	51.4%	[45.1 – 57.7%]
***AOR (95% CI), comparing:***								
*NEETs with workers (ref)*			*2.23*	*(1.68–2.96)*				
*NEETs with students (ref)*			*2.39*	*(1.73–3.29)*				
*NEETs with workers and students (combined, ref)*			*2.41*	*(1.86–3.12)*				
**Number of partners** ^**a**^**, past year**								
**% (95% CI:)**								
0	21.7%	[19.7,23.9%]	8.4%	[5.4–12.9%]	9.1%	[6.9–11.8%]	29.0%	[23.9–34.7%]
1	50.3%	[47.8,52.7%]	61.8%	[56.7–66.7%]	57.1%	[52.8–61.3%]	44.9%	[40.0–50.4%]
2	12.1%	[10.7,13.8%]	11.8%	[8.8–15.6%]	14.4%	[11.6–17.7%]	11.7%	[8.8–15.4%]
3–4	9.3%	[8.1,10.7%]	9.3%	[6.8–12.7%]	11.6%	[9.3–14.4%]	8.5%	[6.1–11.8%]
**5+**	6.5%	[5.3,8.0%]	8.6%	[6.2–11.8%]	7.8%	[5.6–10.7%]	5.9%	[3.5–9.9%]
***AOR (95% CI), comparing:***								
*NEETs with workers (ref)*			*1.13*	*(0.68–1.87)*				
*NEETs with students (ref)*			*1.52*	*(0.74–3.10)*				
*NEETs with workers and students (combined, ref)*			*1.46*	*(0.90–2.37)*				
**% (95%CI) condomless sex with 2+ partners, past year**	7.7%	[6.6 – 9.0%]	11.1%	[8.5–14.5%]	8.9%	[6.7–11.6%]	5.9%	[4.1–8.5%]
***AOR (95% CI), comparing:***								
*NEETs with workers (ref)*			*1.32*	*(0.86–2.03)*				
*NEETs with students (ref)*			*2.04*	*(1.28–3.25)*				
*NEETs with workers and students (combined, ref)*			*1.74*	*(1.19–2.53)*				
**% (95%CI) same-sex experience with genital contact, ever**	7.6%	[6.4 – 8.9%]	10.8%	[8.1–14.2%]	7.9%	[6.0–10.3%]	9.3%	[6.4–13.2%]
***AOR (95% CI), comparing:***								
*NEETs with workers (ref)*			*1.37*	*(0.89–2.11)*				
*NEETs with students (ref)*			*1.24*	*(0.70–2.22)*				
*NEETs with workers and students (combined, ref)*			*1.46*	*(0.97–2.19)*				
Characteristics of sex with most recent partner (MRP)								
**% (95%CI) condom not used on 1st occasionwith most recent partner (vaginal/anal sex only)**	33.7%	[31.2 – 36.3%]	41.8%	[36.7–47.2%]	33.3%	[29.1–37.7%]	31.7%	[25.7–38.3%]
***AOR (95% CI), comparing:***								
*NEETs with workers (ref)*			*1.46*	*(1.09–1.96)*				
*NEETs with students (ref)*			*1.71*	*(1.23–2.38)*				
*NEETs with workers and students (combined, ref)*			*1.57*	*(1.21–2.04)*				
**% (95%CI) just met MRP when first had sex**	3.1%	[2.3 – 4.1%]	4.3%	[2.7–6.9%]	3.1%	[1.9–5.1%]	4.0%	[1.9–8.5%]
***AOR (95% CI), comparing:***								
*NEETs with workers (ref)*			*1.41*	*(0.70–2.83)*				
*NEETs with students (ref)*			*1.35*	*(0.52–3.54)*				
*NEETs with workers and students (combined, ref)*			*1.53*	*(0.81–2.88)*				
	19.0%	[16.9,21.2%]	19.1%	[15.0,23.9%]	17.2%	[14.0, 21.0%]	22.0%	[17.0,27.8%]
	16.1%	[14.3,18.2%]	13.0%	[9.9,16.9%]	14.0%	[11.2,17.6%]	15.2%	[11.7,19.4%]
	12.3%	[10.7,14.0%]	9.0%	[6.6,12.2%]	11.7%	[8.9,15.1%]	13.8%	[10.5,17.9%]
	46.4%	[43.8,49.1%]	48.5%	[43.3,53.8%]	50.2%	[45.9,54.5%]	44.6%	[38.6,50.7%]
	6.3%	[5.2,7.6%]	10.5%	[7.8–13.9%]	6.9%	[5.3–8.9%]	4.5%	[2.5–8.1%]
Sexual health outcomes								
**% (95%CI) Diagnosed with STI, past year**	3.6%	[2.7 – 4.8%]	4.5%	[2.2 – 8.8%]	4.9%	[3.3 – 7.1%]	2.2%	[1.1 – 4.3%]
***AOR (95% CI), comparing:***								
NEETs with workers (ref)			0.93	(0.39–2.21)				
NEETs with students (ref)			1.70	(0.67–4.35)				
NEETs with workers and students (combined, ref)			1.22	(0.57–2.63)				
**% (95%CI) Unplanned pregnancy, past year**	2.6%	[2.0 – 3.4%]	5.1%	[3.4 – 7.6%]	2.8%	[1.7 – 4.7%]	1.6%	[0.8 – 3.2%]
***AOR (95% CI), comparing:***								
NEETs with workers (ref)			1.91	(0.97–3.78)				
NEETs with students (ref)			3.40	(1.49–7.76)				
NEETs with workers and students (combined, ref)			2.64	(1.44–4.84)				
**% (95%CI) Experienced attempted non-volitionalsex, ever**	20.4%	[18.3 – 22.6%]	24.0%	[19.5 – 29.1%]	18.3%	[15.2 – 21.9%]	25.6%	[20.5 – 31.6%]
***AOR (95% CI), comparing:***								
NEETs with workers (ref)			1.46	(1.03–2.07)				
NEETs with students (ref)			1.04	(0.70–1.53)				
NEETs with workers and students (combined, ref)			1.33	(0.97–1.81)				
**% (95%CI) Experienced completed non-volitionalsex, ever**	8.5%	[7.2 – 10.1%]	11.2%	[8.3 – 15.0%]	7.0%	[5.1 – 9.5%]	13.6%	[9.4 – 19.1%]
***AOR (95% CI), comparing:***								
NEETs with workers (ref)			1.71	(1.05–2.76)				
NEETs with students (ref)			1.03	(0.60–1.79)				
NEETs with workers and students (combined, ref)			1.49	(0.99–2.23)				
**% (95%CI) Distressed/worried about sex life**	10.1%	[8.7 – 11.8%]	13.3%	[9.9 – 17.5%]	9.1%	[6.9 – 11.9%]	13.3%	[9.3 – 18.7%]
***AOR (95% CI), comparing:***								
NEETs with workers (ref)			1.50	(0.97–2.32)				
NEETs with students (ref)			1.15	(1.01–1.23)				
NEETs with workers and students (combined, ref)			1.39	(0.94–2.05)				
Health seeking behaviours								
**% (95%CI) Attended sexual health clinic, past year**	22.4%	[20.1 – 24.8%]	22.3%	[17.8 – 27.6%]	24.8%	[21.1 – 28.8%]	20.5%	[15.7 – 26.3%]
***AOR (95% CI), comparing:***								
NEETs with workers (ref)			0.96	(0.62–1.48)				
NEETs with students (ref)			0.67	(0.41–1.10)				
NEETs with workers and students (combined, ref)			0.86	(0.57–1.31)				
**% (95%CI) Blood test for HIV, past year**	12.3%	[10.7 – 14.2%]	17.5%	[13.2 – 22.7%]	15.8%	[12.9 – 19.3%]	9.2%	[6.1 – 13.7%]
***AOR (95% CI), comparing:***								
NEETs with workers (ref)			1.18	(0.77–1.80)				
NEETs with students (ref)			1.84	(1.13–3.02)				
NEETs with workers and students (combined, ref)			1.46	(1.00–2.13)				
**% (95%CI) Chlamydia test, past year**	54.3%	[51.5 – 57.0%]	57.2%	[51.7 – 62.5%]	55.8%	[51.5 – 60.1%]	52.6%	[45.7 – 59.4%]
***AOR (95% CI), comparing:***								
NEETs with workers (ref)			1.10	(0.82–1.48)				
NEETs with students (ref)			1.19	(0.85–1.67)				
NEETs with workers and students (combined, ref)			1.21	(0.93–1.58)				
**% (95%CI) Emergency contraception use, past year**	6.8%	[5.5 – 8.5%]	6.5%	[4.1 – 10.2%]	5.7%	[3.8 – 8.3%]	7.4%	[4.7 – 11.6%]
***AOR (95% CI), comparing:***								
NEETs with workers (ref)			1.20	(0.60–2.38)				
NEETs with students (ref)			0.78	(0.41–1.47)				
NEETs with workers and students (combined, ref)			1.00	(0.57–1.75)				

Abbreviations: unwt, unweighted; wt, weighted

% and 95% CIs are age standardised

Age-adjusted odds ratio for reporting the responses in bold font (for those variables with > 2 response options) relative to workers, relative to students and relative to both workers and students combined

^a^ Opposite and/or same sex partners

### Sexual health outcomes

Among men, there were no differences in the sexual health outcomes studied (Tables 5 & 6). However, NEET women were more likely than students to report an unplanned pregnancy (past year; AOR = 3.40; Table 5). NEET women were also more likely than workers to have experienced non-volitional sex (AOR = 1.46 and AOR = 1.71, attempted and completed respectively). For women, there was a weak association between reporting distress/worry with sex life and being NEET vs. being employed.

### Clustering of harmful health behaviours and poor health outcomes

**Fig. 1:**
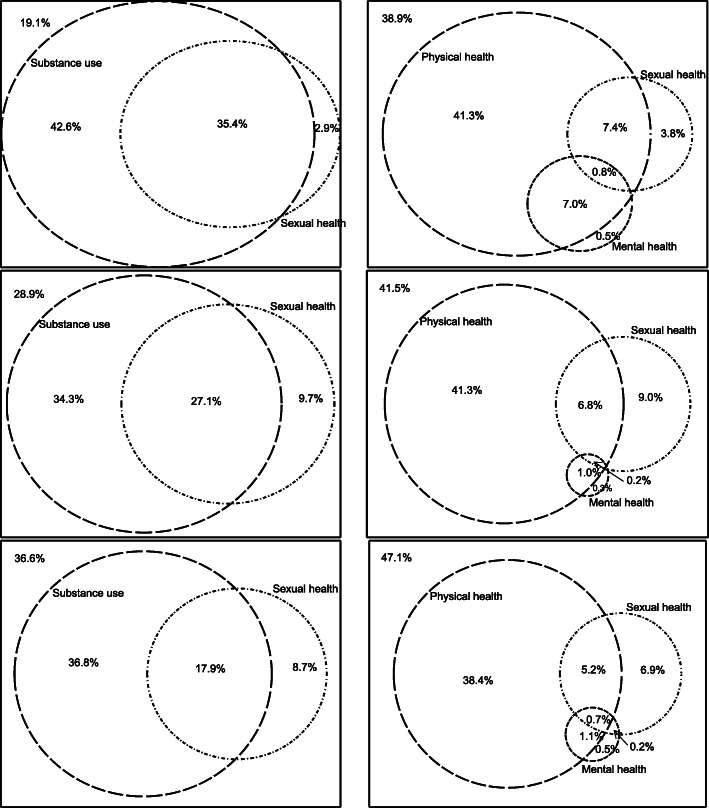
The extent to which harmful health behaviours (left) and poor health outcomes (right) cluster across health domains for males who are NEET (top), workers (middle) and students (bottom)

NEET men were slightly more likely to report multiple harmful physical/mental health behaviours than workers and much more likely than students (Additional file 4; AOR = 1.48 [1.02–2.14] vs. workers and AOR = 2.17 [1.47–3.22] vs. students). They were also more likely to report multiple sexual risk behaviours relative to students (Additional file 4; AOR = 1.88 [1.03–3.43]), and risk behaviours *across* the physical and sexual health domains with 35.4% (29.1–42.3) of males not in employment, education or training reporting at least one harmful physical and sexual health behaviour vs. 27.1% of workers (23.1–31.4) and 17.9% (13.9–22.7) of students (Fig. 1). With respect to poor health outcomes, men who were NEET were also more likely to report multiple poor physical health outcomes than workers (Additional file 4; AOR = 1.87 [1.08–3.21]) and poor health outcomes across more than one domain (Fig. 1; 15.5% [11.1–21.1%] vs. 8.0% [5.8–10.9%] of workers and 7.2% [4.8–10.7%] of students), but there were no differences in the proportion reporting poor health outcomes from all three domains – physical, sexual and mental health (Fig. 1).

**Fig. 2:**
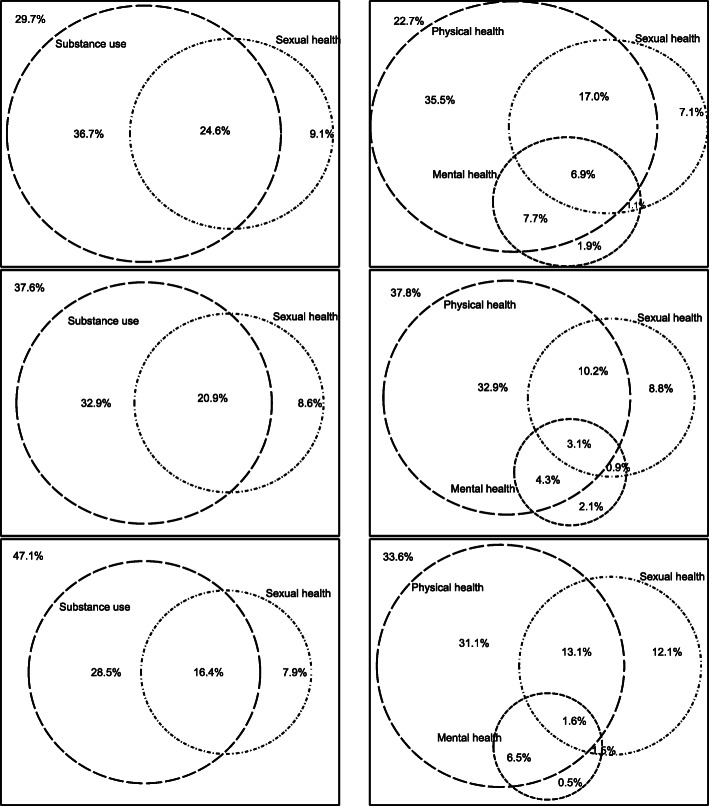
The extent to which harmful health behaviours (left) and poor health outcomes (right) cluster across health domains for females who are NEET (top), workers (middle) and students (bottom)

In contrast, there were no differences for women in the reporting of risk behaviours either *within* or *across* domains for NEETs vs. students or workers (Additional file 5 and Fig. 2). However, women who were NEET were more likely than students or workers to report multiple poor physical health outcomes vs. workers (AOR = 2.12 [1.59–2.82]; Additional file 5) and vs. students (AOR = 1.84 [1.36–2.50]; Additional file 5). They were also more likely to report poor health outcomes across multiple domains (Fig. 2): 32.8% (27.5–38.6%) vs. 8.4% (15.2–22.1%) of workers; and 22.7% (17.7–28.8%) of students. Across all three domains, 6.9% (4.6–10.2%) of women who were NEET reported poor outcomes vs. 3.1% (1.9–4.9%) of workers and 1.6% (0.7–3.5%) of students (Fig. 2).

Looking in detail at the women who were NEET revealed that non-carer NEET women were more likely than NEETs who were carers to report multiple harmful health behaviours (AOR = 2.30 (1.30–4.09)) and poor physical health outcomes (AOR = 2.47 (1.42–4.30)). They were also more likely to report poor outcomes across multiple domains (44.5% (35.5–53.9%) vs. 25.9% (20.5–32.3%)) but there were no substantial differences across all three domains.

## Discussion

### Principal findings

In this study of a nationally-representative sample of young adults, we found that young people who were NEET appear to be in poorer general and mental health. Although women who were NEET take no more sexual risks than their non-NEET counterparts, they appear to experience worse sexual health outcomes. On the other hand, men who are NEET take greater sexual risks than their male counterparts but do not experience any additional detrimental consequence. Looking at health more broadly across different domains, we found that risk behaviours clustered in NEET men but not in NEET women, but that both men and women who were NEET experienced greater clustering of poor health outcomes.

### Strengths and limitations

The main strength of our study is that it uses population-based survey data, and so the results are generalizable to the resident British population when the data were collected. The response rate is in line with other major surveys conducted around this time [30, 31]. Furthermore, through drawing on syndemic theory, which discusses the potential for poor health and behaviours to compound one another, particularly within a social context of inequality [24], this paper has attempted to take a more holistic view of health, considering health across a number of different domains, as well as overlaps between these domains.

Our categorisation of workers, students and NEET was limited by the data available, although we observed a similar NEET prevalence to official statistics [27]. The NEET population is heterogeneous with subgroups that have different characteristics and needs, presenting challenges for policy making and research [11, 32]. We used NEET status as a proxy for vulnerability, but there are limitations in doing so. For example, in our sample, around half of NEET women were looking after children, whereas 70% of NEET men were unemployed. As such, being NEET does not automatically imply vulnerability, and similarly, there will be other vulnerable young adults who are not classified as NEET, e.g. those in temporary/insecure work or those under-employed. Relatedly, NEET status is often temporary and the NEET population fluctuates greatly over time, with some people being NEET for only short periods – complexities a cross-sectional study cannot capture. These classificatory challenges reduce our ability to identify distinct aspects of NEET status and find associations, aside from our survey data preventing us from understanding the direction of these associations and disentangling the mechanisms underlying them. Finally, because of the challenges in capturing social class in young people [33] we have not adjusted for this in our analyses. We observed a strong association between parents’ social class and NEET status. However, we were unable to adjust for this marker of prior disadvantage due to the large quantity of missing data for this variable. We do not therefore know to what extent our associations are a reflection of who *becomes* NEET or the NEET status itself adding to an individual’s background risk.

### Comparison with other studies

Similar to other studies, we found associations between NEET status and family disadvantage, as well as substance use [34, 35] and having a health problem or disability [16] and depression [34]. We also found poorer general health behaviours in young people who were NEET as in a study of young people in England and Scotland [36]. Our study demonstrated that physical/mental health inequalities exist for both men and women, while differences in sexual health outcomes were observed only for women. An Irish study comparing students and non-students (19–22 years) [37] found similar results to ours: no difference in STI diagnosis but higher partner numbers and inconsistent condom use in non-students. However, one study of young women (13–19 years) attending sexual health services reported a higher proportion of women with NEET status than national data (8% vs. 2%), and the women who were NEET reported more sexual partners, earlier first sex and were more likely to report previous STI diagnoses [38]. This inconsistency may reflect differences in age range since students are more likely to attend clinics. However, the differences we found in sexual behaviours are less substantial than those observed in early adolescence. Attitude to school and educational expectations/attainment are strong predictors of teenage pregnancy and other adverse sexual health outcomes in early/mid adolescence [39] and internationally at both a country- and individual-level, girls’ years in education is strongly negatively correlated with fewer adolescent pregnancies [9].

### Meaning of study and implications

Our study was conducted in 2010–2012 when NEET prevalence peaked (at 17% vs. 11% in 2017 [12]), so our population of young people who are NEET may have been a less distinctive group with respect to prior disadvantage than may have previously been seen. Nevertheless, we have demonstrated the poorer health profiles of those who are NEET, with more clustering of poor health outcomes across and within domains than for other young people. This clustering is particularly apparent for females who are NEET who, in addition, are more likely to experience poor sexual and reproductive health outcomes, than students or workers. Furthermore, longer-term economic impacts of the COVID-19 pandemic are likely to increase the proportion of young people who are NEET, and are likely to disproportionally affect the most vulnerable, exacerbating existing inequalities and converging with other impacts of the pandemic for example on mental health.

These findings suggest that there is a group of NEET young people who would benefit from approaches to address health holistically in order to improve overall well-being. Opportunities exist for more joined-up approaches across the sectors (sexual health, primary care, employment training etc.). Our findings also highlight the need for holistic health promotion in this group, and young people more broadly, since risk behaviours were seen to cluster for men. Such clustering of risk behaviours was not observed for women, which may represent gender differences in the main NEET sub-population (unemployed for men vs. carers for women), and that NEET women who were not carers were more likely than NEET women with caring responsibilities to report multiple health risk behaviours. The relationship between mental health problems (which were more common in both NEET men and women), substance use and NEET status is complex and multidirectional. Supporting those with mental health or substance use problems in a timely way may also help prevent young people becoming NEET since prior mental health problems and substance use are associated with NEET status [40].

### Future research

This study’s data are cross-sectional so future research would benefit from taking a longitudinal perspective, both to address causality and reflect how early adulthood is a time of social and sexual transition. Such research could approach analysis using a syndemic approach and should also seek to identify factors that may confer resilience to the experience of NEET status. Future work should seek to understand whether, and to what degree, NEET status deepens prior disadvantage. Qualitative research may help with this, as well as expanding our understanding of the complex needs, behaviours, and outcomes of different subgroups of NEETs, which may not be apparent in a broad quantitative analysis.

## Conclusions

NEETs are of policy interest due to changes in the labour market and as they are at greater risk of social exclusion and adverse health, especially for the minority within this group with complex health needs spanning physical, sexual and mental health domains. These should be tackled in a holistic way to prevent further compounding of inequalities over the life course.

## Supplementary Information

**Additional file 1.** Demographic characteristics of 16–24 year old men and women overall and by education/employment status.

**Additional file 2.** Composition of NEET group.

**Additional file 3.** Description of variables used in the paper.

**Additional file 4.** The extent to which harmful health behaviours (left) and poor health outcomes (right) cluster within health domains for males who are NEET, workers and students.

**Additional file 5.** The extent to which harmful health behaviours (left) and poor health outcomes (right) cluster within health domains for females who are NEET, workers and students.

## Data Availability

The dataset supporting the conclusions of this article is available in the UK Data Service repository, unique persistent identifier: 10.5255/UKDA-SN-7799-2; 10.5255/UKDA-SN-7799-2.

## Abbreviations

AOR: age-adjusted odds ratio
CAPI: computer-administered personal interview
CASI: computer-assisted self-interview
Natsal-3: Third British National Survey of Sexual Attitudes and Lifestyles
NEET: Not in education, employment or training
