# Supplementary material for: How does the sexual, physical and mental health of young adults not in education, employment or training (NEET) compare to workers and students?
Source: BMC Public Health. 2021 Feb 26;21:412. doi: 10.1186/s12889-021-10229-6 (PMC7908525; doi:10.1186/s12889-021-10229-6)
Supplement: Supplementary file 3 — Additional file 3. Description of variables used in the paper. [file 12889_2021_10229_MOESM3_ESM.docx]

**Additional file 3: Description of variables used in the paper**

| **Variable** | **Definition of measure used in analysis** | **How the variable was measured** |
| --- | --- | --- |
| **Demographic variables** |  |  |
| Ethnicity | Participants were grouped as:  1.White if their response was A  2.Asian if their response was C  3. Black if their response was D  4. Mixed/other if their response was B or E | Participants were asked about which ethnic group they belong to. They could choose:  **A. White**  **1.** British  **2.** Irish  **3.** Any Other White background  **B. Mixed**  **4.** White and Black Caribbean  **5.** White and Black African  **6.** White and Asian  **7.** Any Other Mixed background  **C. Asian or Asian British**  **8.** Indian  **9.** Pakistani  **10.** Bangladeshi  **11.** Any Other Asian background  **D. Black or British Black**  **12.** Caribbean  **13.** African  **14.** Any Other Black background  **E. Chinese or other ethnic group**  **15.** Chinese  **16. Any Other** |
| Parent’s social class | Participants responses were classified as:   1. Managerial or professional occupations 2. Technical or skilled occupations 3. Partly-skilled or unskilled occupations 4. Hasn’t had a job 5. Missing data | Several questions based on the International Standard occupation codes were asked:  *See the following website for the International Standard Occupation Codes (SOC) for details: http://www.ons.gov.uk/ons/guide-method/classifications/archived-standard- classifications/standard-occupational-classification-2000/index.html* |
| Quintile of Index of Multiple Deprivation | Participants who were in the highest quintile were treated as living in a ‘most’ deprived area. | Postcodes were used to obtain IMD scores. The adjusted IMD score was generated using a method by Payne and Abel.^[[1]](#footnote-1)^ It is based on the income and employment of the individual country IMD scores combined with the co-efficients and residual values from a linear regression of income and employment on the overall IMD score for each country. The combined scores were generated using the most up-to-date scores for each country at the time. These were IMD 2010 for England, IMD 2011 for Wales, and IMD 2009 for Scotland. |
| Household structure | Participants were grouped as follows depending on the answers to these questions:  Lives Alone  Lives with Partner and/or Children  Lives with Parents  Lives with Non-relatives  Other | Participants were asked:  Including yourself, how many people live here regularly as members of this household. INTERVIEWER: Enter number of people INCLUDING respondent.  Then, for each person:  What was (*name*)’s age last birthday?  How is (*name*) related to you?  1. Spouse / civil partner  2. Cohabiting partner  3. Son/daughter (incl. adopted)  4. Step-son/daughter  5. Foster child  6. Son-in-law/daughter-in-law  7. Parent/guardian  8. Step-parent  9. Foster parent  10. Parent-in-law  11. Brother/sister (incl. adopted)  12. Step- or half-brother/sister  13. Foster brother/sister  14. Brother/sister-in-law  15. Grand-child  16. Grand-parent  17. Other relative  18. Other non-relative (e.g. flat mates) |
| Relationship status | Participants were grouped as:  1: Married / civil partnership or living with partner *if* response was 1, 2 or 3  2: In a steady relationship but not living together *if* response was 4  4: Not in a steady relationship if response was 5, 6, 7 or 8 | Participants were asked:  At present are you  1. single, that is never married and never registered in a same-sex civil partnership  2. married and living with husband/wife  3. in a registered same-sex civil partnership and living with your partner  4. separated, but still legally married  5. divorced  6. widowed  7. [spontaneous only] separated, but still legally in a same-sex civil partnership  8. [spontaneous only] formerly a same sex civil partner, the civil partnership now legally  dissolved  9. [spontaneous only] a surviving civil partner: his/her partner having since died |
| **Health behaviours & profiles** | | |
| Current smoker | Participants who said ‘yes’ to smoking cigarettes at all ‘nowadays’ were treated as current smokers | Participants were asked:  Do you ever smoke cigarettes at all nowadays?"  (Includes roll-ups but excludes cigars).  1. Yes  2. No |
| Average alcohol consumption per week | Questions on frequency of drinking and number of units drunk were combined to determine average alcohol consumption per week. | Participants were asked:  Do you ever drink alcohol nowadays?  1. Yes  2. No  IF Yes THEN  How often have you had an alcoholic drink of any kind during the last 12 months?  1. Five or more days a week  2. Three or four days a week  3. Once or twice a week  4. Once or twice a month  5. Once or twice in the last 12 months  6. Not at all in the last 12 months  IF Once or twice a month or more THEN  This card shows what we mean by units of alcohol. About how many units do you usually have on the days when you have any. Please don’t count special occasions?  1. One or two  2. Three or four  3. Five or six  4. More than six  5. I Only drink on special occasions  6. Other answer |
| Drug use in last year | Participants who reported using any of the listed recreational drugs including injecting drugs in the last 12 months were treated as recreational drug users in the last year | If they had ever used taken:  1. cannabis (marijuana, grass, hash, ganja, draw, skunk, weed, spliff);  2. amphetamines (speed, whizz, uppers, billy);  3. cocaine or coke (charlie), crack (rock, stones, white);  4. ecstasy (E); heroin that was not injected (smack, skag, H, brown, gear, horse);  5. acid or LSD (tabs, trips) or magic mushrooms;  6. crystal Meth;  7. amyl Nitrates (poppers, liquid gold, rush);  8. other non-prescribed drugs or none of these.  If yes to any of these they were asked:  Have you taken (name of drug) in the last 12 months?  1. Yes  2. No |
| Self-reported health status | Participants reporting their health was fair, bad or very bad were compared to those reporting good or very good health | Participants were asked:  How is your health in general? Would you say it is ...READ OUT...  1. very good,  2. good,  3. ...fair,  4. ...bad,  5. or very bad? |
| Long-standing illness, disability or infirmity | Participants who said yes to this question | Participants were asked:  "Do you have any long-standing illness, disability or infirmity? By long-standing I mean anything that has troubled you over a period of time, or that is likely to affect you over a period of time?  1. Yes  2. No |
| 1+ chronic health condition | Yes to any of the following conditions: arthritis, heart attack, coronary heart disease, angina, other forms of heart disease, hypertension, stroke, diabetes, broken hip or pelvis bone or hip replacement ever, backache lasting longer than 3 mo, any other muscle or bone disease lasting longer than 3 mo, treatment for depression, treatment for cancer, and treatment for any thyroid condition in the past year | Participants were asked:  Has a doctor ever told you that you have any of the medical conditions listed on this card?  1 Yes  2 No  If Yes they were asked:  Which ones?  1 Arthritis  2 Heart attack  3 Coronary Artery disease / angina / other form of heart disease  4 Hypertension, also known as high blood pressure  5 Stroke  6 Diabetes, also known as high blood sugar  7 Chronic Lung disease (not including asthma)  8 Parkinson’s disease  9 Epilepsy  They were also asked:  In the last 12 months, that is since (date 12 months ago), have you received treatment from a health professional for any of the medical conditions listed on this card?  1 Yes  2 No  If yes, they were also asked:  Which ones in the last year?  1 Back ache lasting for 3 months or longer  2 Any other muscle or bone disease lasting for 3 months or longer  3 Depression  4 Any other mental health condition  5 Any other neurological condition, apart from Parkinson’s disease and epilepsy  6 Cancer  7 Any thyroid condition  8 Any (ovarian/testicular) or pituitary condition |
| Body mass index |  | Calculated from self reported height and weight |
| Treated for depression in the past year | Answering yes to treatment in the past year and then mentioning option 3, depression | Participants were asked:  In the last 12 months, that is since (date 12 months ago), have you received treatment from a health professional for any of the medical conditions listed on this card?  1 Yes  2 No  If yes, they were also asked:  Which ones in the last year?  1 Back ache lasting for 3 months or longer  2 Any other muscle or bone disease lasting for 3 months or longer  3 Depression  4 Any other mental health condition  5 Any other neurological condition, apart from Parkinson’s disease and epilepsy  6 Cancer  7 Any thyroid condition  8 Any (ovarian/testicular) or pituitary condition |
| **Sexual behaviour** | | |
| First heterosexual sex before the age of 16 | Those reporting an age of <16 were defined as having had sex before the age of 16 | Participants were asked:  How old were you when you first had sexual intercourse with someone of the opposite sex, or hasn't this happened? |
| Not sexually competent at sexual intercourse | First intercourse was classified as competent if there was absence of duress and regret about timing; if there was autonomy of decision; and if a reliable form of contraception was used. (i.e., if participants responses were those that are highlighted in bold then they were treated as being sexually competent). If any responses were not those in bold then first intercourse was classified as not competent. | Constructed variable to capture readiness, combining consensuality, autonomy of decision making, timing, and use of effective contraception.  Participants were asked:  Would you say that you were both equally willing to have intercourse that first time, or was one of you more willing than the other?  IF ONE MORE WILLING: Who was more willing?  1. **Both equally willing**  2. Respondent more willing  3. Partner more willing  Did you or your partner use any form of contraception or take any precautions that first time, or not? (Code all that apply)  **1. Condom (Sheath/Durex)**  **2. The Pill**  3. Emergency contraception  4. Other contraception  5. (partner) withdrew  6. Made sure it was a safe period  7. No precautions by me, don't know about partner  8. No precautions by either of us  Looking back now to the first time you had sexual intercourse, do you think…  1. ...you should have waited longer before having sex with anyone,  2. or, that you should not have waited so long,  **3. or, was it about the right time?**  Which of these things applied to you at the time? (Code all that apply).  **1. I was curious about what it would be like**  **2. I was carried away by my feelings**  *3. Most people in my age group seemed to be doing it*  4. **It seemed like a natural 'follow on' in the relationship**  *5. I was a bit drunk at the time*  *6. I had smoked some cannabis*  *7. I had taken some other drugs*  **8. I wanted to lose my virginity**  **9. I was in love**  10. Other particular factor (SPECIFY AT NEXT QUESTION)  11. Can't remember |
| Number of partners, past year | The total number of opposite sex and same sex partners participant reported in the past year. | And altogether, in the last year, how many (*women/men*) have you had sexual intercourse with? Please type in the number in the last year, '0' if none.  And - altogether, in the last YEAR, how many (men/women) have you had sex with? Please type in the number in the last year, '0' if none.  Answers to these two questions were added together to give total partners in the past year. |
| Condomless sex with 2+ partners, past year | Answers to these questions were used to determine how many partners (opposite or same sex) a participant had had sex with without a condom in the past year. | Participants were asked:  In the last YEAR have you ever had vaginal (or anal) intercourse with a (woman/man) without using a condom?  1. Yes (have had intercourse without a condom in the past year)  2. No (have used a condom on all occasions of vaginal (or anal) intercourse in the past year)  IF Yes THEN  How many (women/men) have you had vaginal (or anal) intercourse with in the past year without using a condom?  Men were asked:  In the last year, when you've had anal sex, how often have you, or your partner, used a condom?  1. Every time  2. Most of the time  3. Occasionally  4. Not at all in the last year  IF Most of the time to not at all in the past year THEN  In the last year, with how many men have you had anal intercourse without using a condom? |
| Same-sex experience with genital contact, ever | Participants who responded as yes were treated as having had ‘any same-sex experience involving genital contact’ | Participants were asked: Have you had sex with a (same sex partner mentioned depending on sex of the participant) involving (genital area/penis/vaginal) contact? (That is oral (or anal) sex or any other contact involving the genital area.)  1 Yes  2 No |
| Condom not used on first occasion of sex with most recent partner | Participants who responded no were considered to not have used a condom. | For their most recent partner, participants were asked:  And (*was/did you use*) a condom (*used*) on that first occasion with (*That person/name*)? If you had only oral sex, and not vaginal or anal sex, on the first occasion, please choose answer option 3, even if you did use a condom. 1. Yes  2. No  3. We only had oral sex on the first occasion. |
| Just met most recent partner when first had sex | Participants responding 6 were considered to have just met their most recent partner | For their most recent partner, participants were asked:  Which one of these descriptions applies best to you and (*That person/name*) at the time you first had sex?  1. We were living together as a couple / married / in a civil partnership at the time  2. We were in a steady relationship at the time  3. We used to be in a steady relationship, but were not at that time  4. We had known each other for a while, but were not in a steady relationship  5. We had recently met  6. We had just met for the first time |
| Age difference of 5+ years with most recent partner | From answers to these questions combined with participant’s age, an age difference of 5+ years was calculated | When was the first occasion with (*That person/name*)?  Type in the year at this question.  If first had sex with that partner in the last 5 years Type in the month number. Please estimate the month if you can’t say exactly.  How old was (*That person/name*) (*on the first occasion/when*) you had sex together?  Type in the age in years. Please estimate the age if you can’t say exactly. |
| Duration of relationship with most recent partner |  | When was the most recent occasion you had sex with (That person/name)? Type in the year at this question and the month at the next question. If not sure of the exact month or year please give your best estimate.  If most recent sex with partner less than 5 years ago. Type in the month number. Please estimate the month if you can’t say exactly.    When was the first occasion with (*That person/name*)?  Type in the year at this question.  If first had sex with that partner in the last 5 years  Type in the month number. Please estimate the month if you can’t say exactly. |
| **Sexual health** | | |
| Diagnosed with STI, past year | Participants with a diagnosis with any of the following STIs in the last year: Trichomonas, Gonorrhoea, Chlamydia, Syphilis, NSU/NGU, Genital warts, Herpes | Participants were asked:  Have you ever been told by a doctor or other healthcare professional that you had any of the following?  1. Chlamydia  2. Gonorrhoea  3. Genital Warts (venereal warts)  4. Syphilis  5. Trichomonas vaginalis (Trich, TV)  6. Herpes (genital herpes)  7. Pubic lice / crabs  8. Hepatitis B  9. (Men only:) NSU (Non Specific Urethritis), NGU (Non Gonococcal Urethritis)  10. (Men only:) Epididymitis  11. (Women only:) Pelvic Inflammatory Disease (PID, salpingitis)  12. (Women only:) Vaginal thrush (Candida, Yeast infection)  13. (Women only:) Bacterial vaginosis  14. Yes, but can't remember which  15. None of these  Then for each infection that the participant had chosen they were asked:  When were you last told by a doctor or healthcare professional that you had  (name of infection)?  1. Less than 1 year ago  2. Between 1 and 5 years ago  3. Between 5 and 10 years ago  4. More than 10 years ago |
| Unplanned pregnancy, past year |  | Participants who had been pregnant in the past year were asked a series of questions about the circumstance around their last pregnancy, these were scored according to a validated measure^[[2]](#footnote-2)^ |
| Attempted non-volitional sex | Participants who answered yes to this question | Participants were asked:  Since the age of 13, has anyone tried to make you have sex with them, against your will?  1. Yes  2. No  3. Don’t know |
| Completed non-volitional sex | Participants who answered yes to this question | Participants who answered yes were asked:  And since the age of 13, has anyone actually made you have sex with them, against your will?  1. Yes  2. No  3. Don’t know |
| Distressed/worried about sex life | Participants who strongly agreed or agreed with the statement | Participants were asked:  Thinking about your sex life in the last year, how much do you agree or disagree with the following statements:  “I feel distressed or worried about my sex life.”  1. Agree strongly  2. Agree  3. Neither agree nor disagree  4. Disagree  5. Disagree strongly |
| Attended sexual health clinic, past year | Participants who had been to a clinic in the past year | Participants were asked:  Have you ever attended a sexual health clinic (GUM clinic)?  1. Yes  2. No  IF yes, then when was that?  1. Less than 1 year ago  2. Between 1 and 5 years ago  3. Between 5 and 10 years ago  4. More than 10 years ago |
| Blood test for HIV, past year | Participants who had had an HIV test in the past year | Participants were asked:  Have you ever had a test for HIV (the virus that causes AIDS)?  1. Yes  2. No  3. Maybe/Not sure  IF yes, When was that test? (the last HIV test if more than one)  1. In the last year  2. Between 1 and 2 years ago  3. Between 2 and 5 years ago  4. Longer than 5 years ago |
| Chlamydia test, past year | Participants who had had a Chlamydia test in the past year | Participants were asked:  In the last year, have you been tested for Chlamydia?  1. Yes  2. No |
| Emergency contraceptive use, past year | Women who reported having used ‘morning after pill’ and/or ‘emergency intra-uterine device (IUD)’ in the past year were treated as users of ‘emergency’ contraception. | Participants were asked:  And which have you used at all with a partner in the last year?  (A) No method used in the last year  (B) (Partner has been /I am sterilized)  (C) (I have been /partner has been sterilized (had vasectomy))  (D) The Pill  (E) Male condom  (F)Female condom  (G) Morning after pill  (H) Emergency intra-uterine device (IUD)  (I) Coil/intra-uterine device (IUD)  (J) Hormonal IUD - MIRENA  (K) Cap/diaphragm  (L) Injections  (M) Spermicides (foams/gels/sprays/pessaries)  (N) Natural family planning (safe period/rhythm method/Persona)  (O) Withdrawal  (P) Implants  (Q) Other answer given at previous question |

1. Payne R, Abel G. UK indices of multiple deprivation – a way to make comparisons across constituent countries easier. *Health Statistics Quately* 2012; **Spring 2012**. [↑](#footnote-ref-1)
2. Barrett G, Smith SC, Wellings K Conceptualisation, development, and evaluation of a measure of unplanned pregnancy Journal of Epidemiology & Community Health 2004;58:426-433. [↑](#footnote-ref-2)
